# Viruses and Lipids

**DOI:** 10.3390/v2051236

**Published:** 2010-05-20

**Authors:** Akira Ono

**Affiliations:** Department of Microbiology and Immunology, University of Michigan Medical School, 5736 Medical Science Building II, 1150 W. Medical Center Drive, Ann Arbor, MI 48109-0620, USA; E-Mail: akiraono@umich.edu; Tel.: +1-734-615-4407; Fax: +1-734-764-3562

As obligatory intracellular pathogens, viruses exploit various cellular molecules and structures, such as cellular membranes, for their propagation. Enveloped viruses acquire lipid membranes as their outer coat through interactions with cellular membranes during morphogenesis within, and egress from, infected cells. In contrast, non-enveloped viruses typically exit cells by cell lysis, and lipid membranes are not part of the released virions. However, non-enveloped viruses also interact with lipid membranes at least during entry into target cells. Therefore, lipids, as part of cellular membranes, inevitably play some roles in life cycle of viruses.

Studies of lipids in the virus life cycle, first as components of enveloped viruses, trace back to several decades ago. Since then, however, our understanding of the roles of lipids in cellular functions has evolved dramatically; lipids are no longer thought to be just plain building blocks of membrane bilayers. Some lipids promote molecule compartmentalization through active participation in formation of microdomains/vehicles, whereas others bind specific proteins thereby serving as signaling molecules. With the emergence and establishment of concepts such as lipid rafts and headgroup-specific lipid-protein interactions [1,2], our understanding of relationships between viruses and lipids has also evolved substantially in the recent years. This special issue of Viruses, “**Role of Lipids in Virus Replication**”, brings together reviews by experts of both enveloped and non-enveloped viruses. They discuss the latest views on interactions of viruses with lipids and/or lipid-based structures in various aspects of the virus life cycle, ranging from entry, to genome replication, to assembly and release.

All viruses need to attach to the host cell surface and gain access to the cytoplasm where they replicate. Glycosphingolipids, with diverse sugar chain modifications, serve as virus receptors for a wide variety of viruses. **Taube, Jiang, and Wobus** [3] describe the interactions between various glycosphingolipid species and a large number of non-enveloped viruses, namely calici-, rota-, polyoma- and parvoviruses, and discuss roles played by glycosphingolipids as attachment or entry receptors during entry of these viruses.

**Kielian, Chanel-Vos, and Liao** [4] take us through molecular mechanisms promoting entry of alphaviruses, a group of enveloped positive-sense RNA viruses. It has long been known that alphavirus entry is a cholesterol-dependent process. The structural biology, biochemistry, and cell biology of alphavirus internalization and membrane fusion reviewed here provide a detailed picture of the series of events during these process and insights into roles played by various viral and host determinants, including lipids.

Positive-sense RNA viruses reorganize cellular membranes to form membranous structures with which genome replication complexes associate. However, specific roles played by lipid molecules *per se* have begun to emerge only recently. **Stapleford and Miller** [5] discuss potential roles that lipid constituents of cell membranes play during the formation of these membrane structures and replication of RNA genomes using examples of plant, insect, and mammalian positive-sense RNA viruses.

One of the most clinically relevant positive-sense RNA viruses is hepatitis C virus (HCV). For this virus, the connections between lipid metabolism and RNA genome replication, infectious particle assembly, and entry, are becoming increasingly clear. **Targett-Adams, Boulant, Douglas, and McLauchlan** [6] review the current understanding of the contributions of lipid droplets (cytoplasmic storage organelles consisting of triacylglycerol and cholesterol ester) and biosynthesis of fatty acids and cholesterol to key stages of the HCV life cycle.

Recent data suggest that in some cases non-enveloped viruses exit without lysis of producer cells. **Bhattacharya and Roy** [7] discuss the unexpected similarity between enveloped and non-enveloped viruses, with a particular focus on recent findings in assembly and exit of reoviruses, Bluetongue virus and rotavirus, which interact with lipid rafts during the late phase of life cycle. This review also highlights structural similarities between the viral proteins mediating virus entry of Bluetongue virus and enveloped viruses.

Poxviruses acquire multiple lipid membranes during their replication and assembly in the cytoplasm. Recent studies suggest the importance of anionic phospholipids present in viral membranes for efficient virus entry. **Laliberte and Moss** [8] describe the current understanding of mechanisms by which poxviruses acquire layers of membranes, and discuss the roles played by lipids and membranes in the virus entry process as well as during the assembly, morphogenesis, and egress of progeny virions.

**Waheed and Freed** [9] describe a wide variety of roles that lipids play during the life cycle of retroviruses. Retroviruses have been observed to associate with cholesterol-rich lipid raft microdomains during virus particle assembly and entry. Interactions of viral proteins with specific anionic phospholipid and sphingolipid species have also been shown to regulate or modulate these processes. This review provides current knowledge on the relationships between retroviruses and lipids or lipid microdomains, and discusses the effects of lipid-modifying agents on retrovirus replication.

As summarized above, this special issue provides the latest view of lipid-virus interactions during entry, genome replication, morphogenesis, and exit, for a wide variety of viruses. The theme emerging from the reviews in this issue is that lipids play active roles in the virus life cycle as host cofactors rather than inert constituents of cell membranes. Since the processes lipids facilitate are essential for virus propagation, advances in our understanding in this field also promise new insights into prospects for developing new therapeutic strategies.
